# Impact of a Stroke Same Day Emergency Care Service on MRI Wait Times and Patient Flow in Mild Stroke Presentations

**DOI:** 10.7759/cureus.96785

**Published:** 2025-11-13

**Authors:** Wei Jia Liu, Roy Wang, Lorraine Lemke, John Kamara, Richard Marigold

**Affiliations:** 1 Department of Stroke, University Hospital Southampton NHS Foundation Trust, Southampton, GBR

**Keywords:** brain magnetic resonance imaging, mild stroke, patient flow optimization, quality improvement project (qip), same day emergency care (sdec), stroke, united kingdom (uk)

## Abstract

Background

With an aging and increasingly comorbid population, stroke services in the United Kingdom face substantial pressure. A significant proportion of admissions under the stroke team involve patients presenting with mild symptoms. Current guidelines recommend urgent MRI for cases with mild symptoms and/or diagnostic uncertainty. A stroke Same Day Emergency Care (SDEC) service is designed to provide rapid investigations, treatment, and discharge for patients presenting with mild stroke symptoms and a low probability of stroke. This study aims to investigate whether a stroke SDEC pathway reduces MRI wait times and hospital admissions among patients presenting with mild stroke symptoms.

Methods

A retrospective study was conducted in the stroke department at a single tertiary center. Mild stroke symptoms were defined as a National Institutes of Health Stroke Scale (NIHSS) score ≤ 4. Patients with NIHSS ≤ 4 were identified from the stroke department referral database over two six-week periods: pre-SDEC (September 23 to November 3, 2024) and during the stroke SDEC trial period (November 4 to December 16, 2024). MRI wait times, diagnoses, and admission rates were analyzed.

Results

During the pre-SDEC period, 73 patients had NIHSS ≤ 4, with a mean MRI wait time of 16 hours and 33 minutes; 86% of these patients were admitted to the stroke unit. During the stroke SDEC trial period, 104 patients had NIHSS ≤ 4, with a mean MRI wait time of 11 hours and 34 minutes; 66% were admitted. Among these, 40 patients with NIHSS ≤ 4 were seen in the stroke SDEC, with a mean MRI wait time of three hours and 50 minutes and an admission rate of 20%. Average MRI wait times across the stroke department for patients with NIHSS ≤ 4 decreased significantly between the pre-SDEC and stroke SDEC trial periods (p = 0.016), alongside a reduction in admission rates (p = 0.00269).

Conclusions

During the stroke SDEC trial period, mean MRI wait times and stroke unit admission rates among patients with mild stroke symptoms were significantly reduced. This approach facilitates rapid diagnosis and treatment while increasing inpatient capacity for stroke patients with more severe deficits and/or those requiring thrombolysis or mechanical thrombectomy.

## Introduction

Stroke is a leading cause of death and long-term disability worldwide [1]. In the United Kingdom, approximately 100,000 people experience a stroke each year, and with an aging and increasingly comorbid population, there is significant pressure on stroke services [2]. A substantial proportion of patients admitted to stroke units present with mild stroke symptoms, generally defined as a National Institutes of Health Stroke Scale (NIHSS) score ≤ 4 [3,4]. While many of these cases represent true strokes, a significant number are stroke mimics or transient ischemic attacks (TIAs). These patients may experience prolonged admissions, largely due to delays in imaging and diagnostic clarification.

The National Optimal Stroke Imaging pathway recommends that, for patients presenting with mild symptoms and/or diagnostic uncertainty, MRI should be performed within one hour of hospital admission [5]. MRI is particularly valuable in detecting small infarcts in patients with mild stroke symptoms, which may be missed on CT [5]. Delayed imaging can hinder patient flow and contribute to prolonged hospital stays [6].

Same Day Emergency Care (SDEC) pathways have been developed across the United Kingdom’s NHS [7,8]. These consultant-led ambulatory services aim to provide rapid clinical assessment and key diagnostics within a single day to improve patient flow and facilitate safe discharge, thereby reducing inpatient pressures. Reviews suggest potential benefits in decreasing length of stay and avoiding unnecessary admissions, although the evidence remains heterogeneous [6]. While SDEC has been implemented across multiple departments and specialties, its impact on stroke services is less well studied. Emerging models, such as the University College London Hospital neurology SDEC, provide ambulatory pathways for acute neurological presentations, enabling same-day imaging and senior review; reports suggest reductions in admissions and hospital length of stay [9].

This study evaluates whether the introduction of a stroke SDEC service improves care for patients with mild stroke symptoms by reducing MRI wait times and stroke unit admissions.

## Materials and methods

Study design

A single-center retrospective study was conducted at a tertiary stroke center in the United Kingdom. The study compared MRI wait times and admission rates across the stroke department for patients presenting with mild stroke symptoms (NIHSS ≤ 4) during a six-week pre-SDEC period (September 23 to November 3, 2024) and a six-week stroke SDEC trial period (November 4 to December 16, 2024). The study was conducted in accordance with institutional ethical standards and the principles of the Declaration of Helsinki. As this work was a local quality improvement project involving a retrospective review of anonymized patient data, formal ethical approval from the NHS Research Ethics Committee was not required.

Stroke SDEC pathway

The stroke SDEC service was designed to provide rapid assessment and care for patients presenting with minor strokes, stroke mimics, or TIAs requiring senior clinical evaluation and MRI as indicated. During the trial period, the stroke SDEC operated alongside existing stroke assessment pathways, and patients were seen either via the stroke SDEC or through existing pathways, such as the hyperacute stroke unit (HASU).

Patients were referred to stroke advanced clinical practitioners (ACPs) from primary care, the emergency department, and paramedics and were subsequently triaged. Patients deemed by the stroke ACP to clearly not have a stroke were redirected to the emergency department, primary care, or another specialty. Patients suspected of having a minor stroke, TIA, or stroke mimic but requiring exclusion of stroke were directed to the stroke SDEC for assessment. Patients believed to have suffered a moderate to severe stroke, or those requiring thrombolysis and/or mechanical thrombectomy with an anticipated inpatient stay of over 24 hours, were admitted to the HASU.

**Figure 1:**
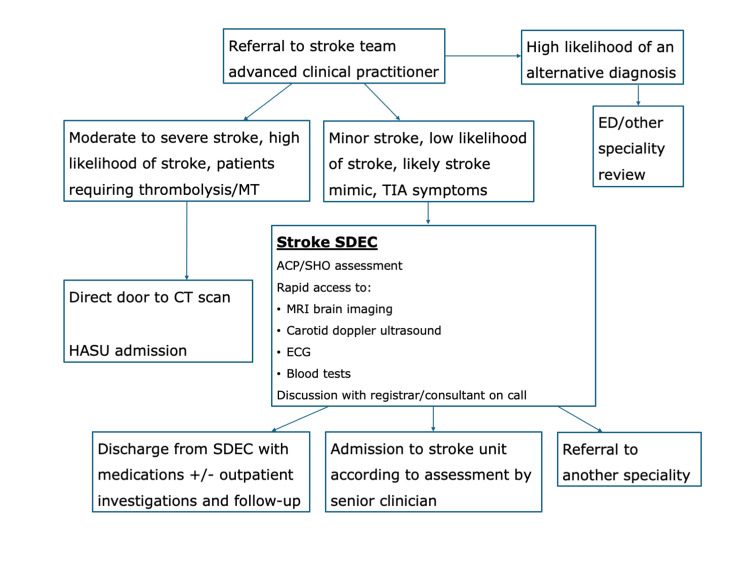
Stroke admission pathway at University Hospital Southampton during the stroke SDEC trial period Flowchart illustrating the stroke service admission pathway from November 4 to December 16, 2024. ACP, advanced clinical practitioner; HASU, hyperacute stroke unit; MT, mechanical thrombectomy; SHO, senior house officer; SDEC, Same Day Emergency Care; TIA, transient ischemic attack

The stroke SDEC was located near the emergency department and operated from 08:00 to 17:00, Monday through Friday. During the trial period, the stroke consultant covering the TIA clinic and/or the liaison stroke consultant supported the SDEC, alongside the stroke registrar and ACPs. One resident doctor from the HASU was assigned to the stroke SDEC during the trial period; no additional staff were specifically recruited for the SDEC. The pathway is illustrated in Figure 1.

Participants

Patients referred to the stroke service were identified via the ACP referral database and included if assessed by the stroke team with adequate documentation, including NIHSS. Exclusion criteria were missing NIHSS or stroke team documentation, as well as patients seen in outpatient and TIA clinics. Mild stroke symptoms were defined as NIHSS ≤ 4, and only patients with NIHSS ≤ 4 were included in the analysis.

Data collection

For each patient, clinical records were retrospectively reviewed. Extracted data included NIHSS score, diagnosis (stroke or other, e.g., TIA and stroke mimics), MRI performance, and wait time from stroke team assessment and admission to the stroke unit and whether the patient was evaluated via the stroke SDEC pathway during the trial period. Data were collected by three independent researchers, anonymized, and entered into Microsoft Excel (Microsoft 365, Version 2502 Build 16.0.18526.20546; 64-bit; Microsoft Corporation, Redmond, WA, USA) for subsequent analysis.

Statistical analysis

MRI wait times were compared between the pre-SDEC and stroke SDEC trial periods. Statistical analyses were conducted using R (Version 2024.12.1+563; R Core Team, 2024). A Shapiro-Wilk test demonstrated that MRI wait time was non-parametric. A Mann-Whitney U test was used to compare MRI wait times between groups. Chi-square analysis was performed to compare admission rates between the pre-SDEC and stroke SDEC trial periods. The significance threshold was set at p < 0.05. 

## Results

Patient cohort and baseline demographics

During the pre-SDEC period, 172 patients were assessed by the stroke department with adequate documentation, of whom 73 had an NIHSS ≤ 4. Among these 73 patients, 52 underwent MRI, and 45% were diagnosed with stroke. During the six-week stroke SDEC trial period, 202 patients were assessed by the stroke team, with 104 patients having an NIHSS ≤ 4. Of these 104 patients, 67 underwent MRI, and 48% were diagnosed with stroke. During the stroke SDEC trial period, 40 patients with an NIHSS ≤ 4 were seen in stroke SDEC (SDEC subgroup); 29 of these underwent MRI, and 35% were diagnosed with stroke.

A Shapiro-Wilk test indicated that age was not normally distributed. A Mann-Whitney U test demonstrated no significant difference in age between patients in the pre-SDEC and stroke SDEC trial periods (pre-SDEC mean 71.4 years vs. stroke SDEC trial period mean 70.3 years, p = 0.596). A chi-square test showed no significant difference in gender distribution between the pre-SDEC and stroke SDEC trial periods (pre-SDEC 46.6% male vs. stroke SDEC trial period 49.0% male, p = 0.747).

MRI wait times

**Figure 2:**
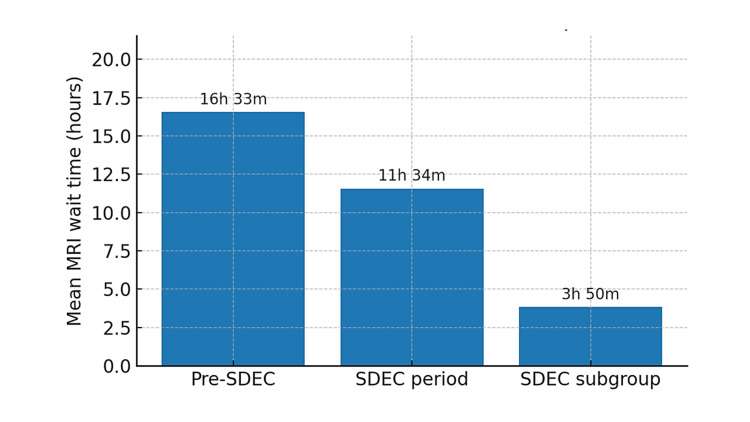
Mean MRI wait times for patients with NIHSS ≤ 4 before and after stroke SDEC implementations Bar chart showing mean MRI wait times across the stroke department for patients with NIHSS ≤ 4 during the pre-SDEC period, the stroke SDEC trial period (SDEC period), and among patients seen in the stroke SDEC (SDEC subgroup). Mean MRI wait times decreased from 16 hours and 33 minutes pre-SDEC to 11 hours and 34 minutes during the stroke SDEC trial period. The mean MRI wait time for the SDEC subgroup was three hours and 50 minutes. SDEC, Same Day Emergency Care; NIHSS, National Institutes of Health Stroke Scale

During the pre-SDEC period, the mean MRI wait time for patients with NIHSS ≤ 4 was 16 hours and 33 minutes across the stroke department. During the stroke SDEC trial period, the mean MRI wait time for patients with NIHSS ≤ 4 was 11 hours and 34 minutes. The average MRI wait time for patients with NIHSS ≤ 4 during the stroke SDEC trial period was significantly shorter than in the pre-SDEC period (p = 0.016). Furthermore, for the patients with an NIHSS ≤ 4 seen in stroke SDEC, the mean MRI wait time was three hours and 50 minutes (Figure 2).

Admission rates

**Figure 3:**
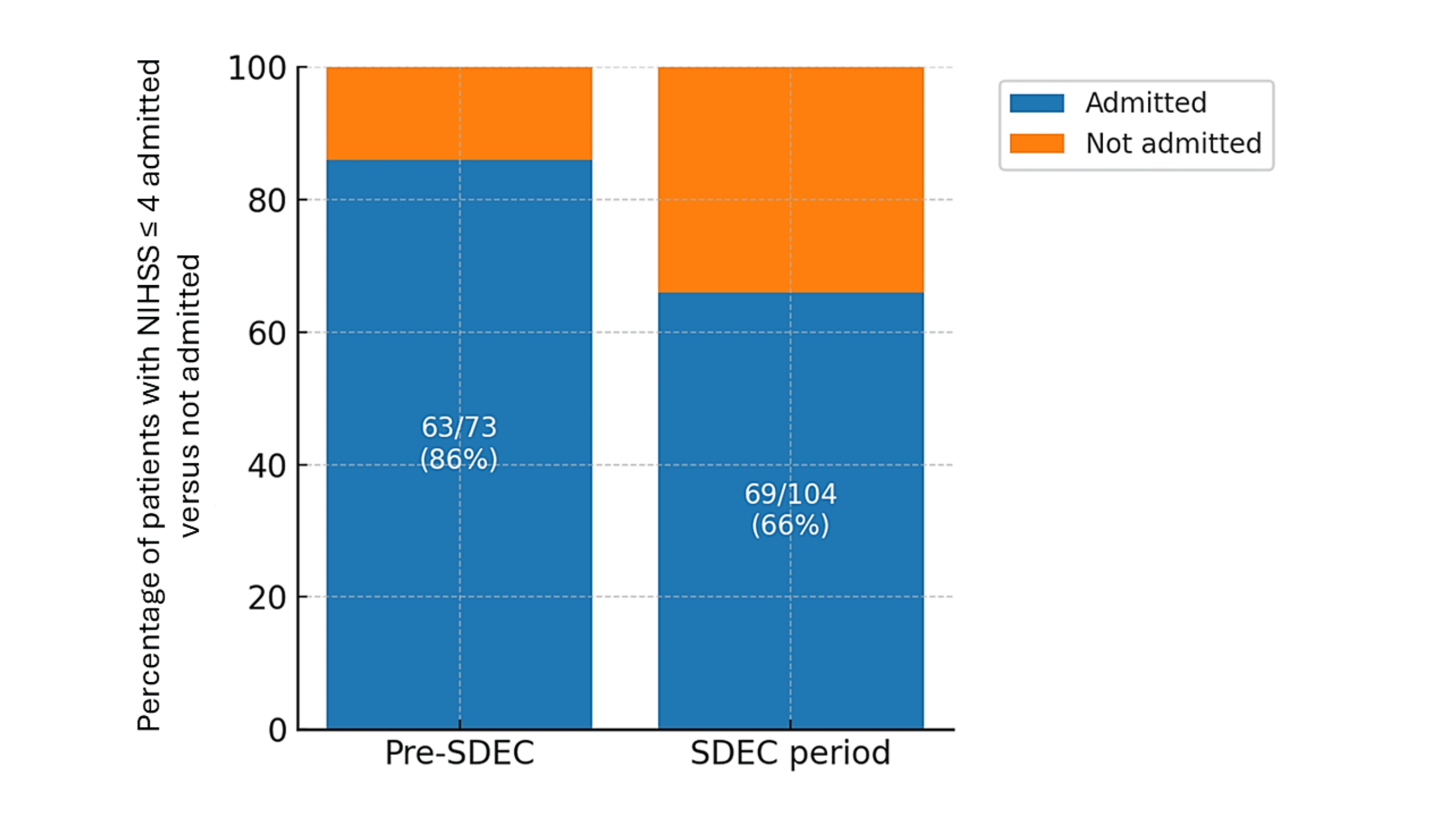
Admission rates to the inpatient stroke unit during the pre-SDEC period and the stroke SDEC trial period for patients with NIHSS ≤ 4 Stacked bar chart showing the proportion of patients with NIHSS ≤ 4 who were admitted (blue) or not admitted (orange) to the inpatient stroke unit during the pre-SDEC and stroke SDEC trial periods. Admission rates decreased from 86% (63/73 patients) in the pre-SDEC period to 66% (69/104 patients) during the SDEC period. SDEC, Same Day Emergency Care; NIHSS, National Institutes of Health Stroke Scale

Before SDEC, 63 of 73 patients (86%) with an NIHSS ≤ 4 were admitted to the stroke unit. During the stroke SDEC trial period, 69 of 104 patients (66%) with an NIHSS ≤ 4 were admitted to the stroke unit (Figure 3). Among patients seen in SDEC, eight of 40 (20%) with an NIHSS ≤ 4 were admitted to the stroke unit. Admission rates among patients with an NIHSS ≤ 4 were significantly lower during the stroke SDEC trial period compared to the pre-SDEC period (p = 0.00269).

## Discussion

The key finding of this study was that during the stroke SDEC trial period, MRI wait times across the stroke department for patients presenting with mild stroke symptoms decreased from a mean of approximately 16.5 hours pre-SDEC to 11.5 hours during the stroke SDEC trial period, facilitating earlier diagnosis. Furthermore, the mean wait time for MRI was under four hours among patients with an NIHSS ≤ 4 seen in the stroke SDEC. A reduction in inpatient admission rates to the acute stroke unit among patients presenting with mild stroke symptoms was also observed during the stroke SDEC trial period.

The significant reduction in MRI wait times in the stroke SDEC likely reflects the targeted structure of the SDEC pathway, including senior clinician triage, prioritization of MRI slots by the radiology department, and streamlined decision-making with high patient turnover. This contrasts with standard inpatient pathways, where imaging queues and bed availability can delay processes [6]. Reduced stroke unit admissions among patients presenting with mild stroke symptoms during the stroke SDEC trial period suggest that early diagnostic clarity may enable ambulatory or outpatient management for lower-risk patients, such as those with stroke mimics. This has resource and bed-management implications, helping preserve stroke unit capacity for patients with higher acuity or treatment needs, such as those requiring thrombolysis and/or mechanical thrombectomy.

The study builds on existing evidence regarding neurology SDEC services in the NHS. Balaratnam et al. trialed a consultant-led neurology SDEC model at University College London Hospital (University College London Hospitals NHS Foundation Trust), which was associated with reduced time to diagnosis and treatment, as well as reduced onward referral rates [10]. Similarly, a study by Hsu et al. at St Mary’s Hospital, London (Imperial College Healthcare NHS Trust), showed that 25% of patients referred to neurology SDEC were discharged after specialist review, thereby reducing inpatient burden [11]. Together with the findings of this study, these results suggest that SDEC models can streamline patient flow and reduce inpatient pressures.

It is well established that imaging delays hinder diagnostic clarity and impact patient flow, often acting as a rate-limiting step in the patient journey. Prabhu et al. demonstrated that even small improvements in imaging throughput can significantly reduce the time a patient spends in acute settings prior to diagnosis and management [12]. This is particularly pertinent in stroke presentations, where delays in diagnosis and treatment can result in significant neurological sequelae [13].

Limitations and future directions 

This study provides real-world data on the effects of a stroke SDEC pathway at a single tertiary center. However, the retrospective design limits the ability to control for confounders, such as variations in staffing and imaging capacity. Data accuracy may also be affected by incomplete or inaccurately recorded documentation. Initial triage and admission to the stroke SDEC were conducted by stroke ACPs, and although this reflects real-life practice, it introduces subjectivity and is practitioner-dependent. In addition, single-institution studies limit generalizability, as resource and service structures may differ significantly elsewhere. This study did not examine patient outcomes such as re-attendance or readmission rates among patients attending the stroke SDEC. Future studies should investigate rehospitalization rates and ideally adopt a multicenter design to assess whether the stroke SDEC model is generalizable across the NHS.

For this study, mild strokes were defined as NIHSS ≤ 4, in accordance with the National Institute for Health and Care Excellence (NICE) guidelines [3]. While NIHSS is a widely used tool for quantifying stroke severity, it has limitations in capturing the complexity of mild strokes. NIHSS is biased toward motor and language impairments and does not adequately cover cognitive deficits, mild visual-spatial neglect, or psychological impacts. It is also less sensitive to posterior circulation strokes compared to anterior circulation strokes [14].

## Conclusions

During the stroke SDEC trial period, average MRI wait times for patients presenting with mild stroke symptoms were significantly reduced, thereby shortening diagnostic delay. Stroke unit admission rates also decreased, potentially freeing inpatient stroke bed capacity for patients requiring thrombolysis, mechanical thrombectomy, or those with more significant deficits. These findings support the role of ambulatory care models in optimizing stroke service delivery. Further research is warranted to assess broader applicability, longer-term patient outcomes, and the economic impact of such models.
